# Patient perceptions of insulin therapy in diabetes self-management with insulin injection devices

**DOI:** 10.1007/s00592-023-02054-7

**Published:** 2023-02-25

**Authors:** Agostino Consoli, Gloria Formoso

**Affiliations:** 1 Department of Medicine and Aging Sciences, Center for Advanced Studies and Technology (CAST, Ex CeSIMet) G. d’Annunzio University Chieti-Pescara, CAST building, Room 315, G. d’Annunzio University Campus, Via Luigi Polacchi, 11-13, 66100 Chieti, Italy; 2 Endocrinology and Metabolism Unit, Pescara Health Service, Pescara, Italy

**Keywords:** Diabetes mellitus, Insulin, Patient preference, Injection device, Connected insulin pen

## Abstract

**Aims:**

Several insulin delivery systems are available to control glycemia in patients with diabetes. Recently introduced devices feature connectivity enabling data transfer to smartphone applications to provide decision support and reduce errors in dosing and timing, while reducing the cognitive burden.

**Methods:**

We conducted an online survey in Italian patients with a self-reported diagnosis of diabetes to assess patient perceptions of insulin therapy management, and their impressions of connection-enabled insulin pens compared to standard insulin pens. The Morisky Medication Adherence Scale-8 was used to assess adherence to insulin therapy.

**Results:**

Among 223 respondents (108 with type 1 diabetes; 115 with type 2 diabetes), the most prominent unmet need was the necessity to overcome the cognitive burden of care associated with measuring, calculating, timing, and recording therapy. Only 25% of respondents had high adherence; 28% had low adherence.

**Conclusions:**

When asked to compare the attributes of a non-connected insulin pen with those of a new connected device, 71% of patients rated the new proposal “very useful”. The cognitive burden associated with self-management of diabetes therapy may influence preferences for advanced insulin delivery systems.

**Supplementary Information:**

The online version contains supplementary material available at 10.1007/s00592-023-02054-7.

## Introduction

Worldwide, over half a billion people (10.5% of the population) have diabetes and projections indicate that the number will increase to 12.2% by 2045 [1]. Self-management plays a key role in treatment success. The complexity of treatment regimens may cause patients to feel overwhelmed by diabetes management [2–4], and non-adherence leading to poor glycemic control is a common problem [5–7].

Convenience and ease of use can influence patient preference [8, 9], and identifying treatment attributes that meet patient preferences has been shown to improve adherence and outcomes [10]. In general, patients tend to prefer simpler treatments [11, 12], and simple interfaces for treatment decision-making support that reduce the cognitive burden of diabetes self-management [13, 14]

Advances in technology have allowed patients to better approximate normal glucose homeostasis through point-of-care or continuous blood glucose monitoring and informatics support for calculating doses and timing [15]. These improvements can optimize insulin delivery and help to overcome problems of sub-optimal management due to errors of mental math, and non-adherence due to missed or mistimed doses.

Connected insulin pens and blood glucose self-monitoring devices transfer data to dedicated smartphone applications that provide dose calculations, send reminders, record injected doses, and integrate information on blood glucose and the time and dose of the previous injection; some systems track meals, physical activity, and insulin treatment [13, 16–18]. Smart connected insulin pens and use of digital technology are associated with improved glycemic control [19–21]; but may also improve the quality of life of people with diabetes by reducing their treatment burden.

We have surveyed patients’ perceptions of insulin therapy in diabetes self-management to understand their unmet needs, assessing the potential of connection-enabled insulin devices to facilitate diabetes self-care and possibly to improve adherence, as well as determining the relative importance they place on features of digitally enhanced insulin delivery devices.

## Methods

The aim of this study was to understand diabetes patients’ levels of satisfaction and unmet needs regarding daily insulin treatment, to investigate the possibility of new insulin pen functions and methods that may improve compliance, and to explore the potential of connection-enabled insulin pens compared to standard insulin pens.

### Survey

#### Qualitative phase

During the qualitative phase, 2 focus groups were conducted to inform development of the final quantitative survey. These explored the impact of insulin therapy on the quality of life of patients with diabetes, assessing their reactions to different profiles of technological devices for insulin therapy, and collected insight regarding the recently introduced Tempo Pen (Eli Lilly Italia S.p.A.).

Each focus group involved 6 adult patients with diabetes, including 2 patients with type 1 diabetes (T1D) receiving multiple daily insulin injections (basal bolus regimen), 2 patients with type 2 diabetes (T2D) receiving basal insulin, and 2 patients with T2D receiving basal bolus insulin. These focus groups were conducted online using the FocusVision platform (InterVu; Forsta, Inc. https://www.forsta.com) and engaged two different macro regions of North and Central-South Italy. Recordings were analyzed by the qualitative researchers.

#### Quantitative phase

Based on the qualitative findings, a survey was designed to investigate patients' perceptions of daily diabetes management and insulin therapy [**Supplemental Material 1**], including satisfaction, difficulties encountered, unmet needs, and the potential of new insulin treatment functions and methods to facilitate treatment management and promote compliance. We also assessed patients’ impressions of connection-enabled insulin pens, as described in a short online video, compared to standard insulin pens. Survey results were summarized as percentages and/or means, and the significance of any differences were analyzed using Student's t-test.

The survey population consisted of adult patients who had been diagnosed with T1D or T2D at least 6 months previously, and who had been receiving basal or basal-bolus insulin therapy for at least 6 months. Eligible respondents were recruited through either computer-assisted web interviewing or a patient panel. Results were interpreted considering patient demographic and disease characteristics, as well as treatment adherence assessed using the Morisky Medication Adherence Scale (MMAS)^1^ [22–24], linguistically validated Italian version [25], to record patient-reported adherence to insulin treatment. The MMAS uses 8 questions to determine adherence behaviors [**Supplemental Material 2]**. The first 7 are yes/no responses, while the last question uses a 5-point Likert scale; adherence is scored using proprietary coding to yield outcomes corresponding to low, medium, or high medication adherence.

## Results

Quantitative interviews were conducted in 223 patients, 108 with T1DM and 115 with T2DM, mean age 47 years. Patients with T1DM all reported receiving basal-bolus therapy, while those with T2DM reported receiving either basal-bolus (56%) or basal therapy (44%). Demographic and clinical characteristics of the population are presented in Table 1.

**Table 1:** Demographic and patient reported clinical characteristics of respondents to the quantitative survey (n = 223)

Attribute	
Age, mean years	47
Women	58%
T1DM, n	108
Disease duration, mean years	13
Treatment duration, mean years	13
Insulin treatment basal-bolus	100%
Monotherapy	98/108 (91%)
Combined with other hypoglycemic agents	10/108 (9%)
T2DM, n	115
Disease duration, mean years	7
Treatment duration, mean years	5
*Insulin treatment*	
Basal-bolus	64/115 (56%)
Monotherapy	46/64 (72%)
Combined with other hypoglycemic agents	18/64 (28%)
Basal	51/115 (44%)
Monotherapy	13/51 (25%)
Combined with other hypoglycemic agents	38/51 (75%)
Employed full-time	165/223 (74%)
Experience managing insulin with an app	96/223 (43%)
*Geographic location in Italy*	
North	123/223 (55%)
South Central	100/223 (45%)
No financial limitations for necessities	176/223 (79%)

### Adherence to therapy

The results from the MMAS-8 scale identified three segments: more than a quarter of patients (28%) had low adherence to insulin therapy and only 25% had a high level of adherence. More patients with high adherence reported being employed full-time, following recommendations for diet and physical activity, and having a stable, predictable lifestyle. Highly adherent patients were also more likely to record their treatment history using pen and paper, a smartphone, or a computer. Overall, patients reported forgetting the timing or dosage of their last injection a mean of 2.4 times per week, and the most frequent reasons given for this were difficulty in respecting instructions and having too much information to manage. More than half of respondents (51%) reported occasionally missing an insulin dose, while 21% reported missing a dose on the previous day.

### Unmet needs that emerged from the survey

The most prominent unmet need highlighted by the survey was the necessity to overcome the cognitive burden of care associated with measuring, calculating, timing, and recording aspects of therapy. Issues mentioned by patients included the amount and complexity of data (82% of mentions), difficulty with following the clinical prescription, and the high level of dosage accuracy required (60% of mentions). Patients report needing support specifically for administering the correct amount of insulin (58%), maintaining blood glucose in their target range (57%), monitoring blood glucose values (53%), and correctly timing insulin administrations (50%).

#### Impressions of connected device attributes

**Fig. 1:**
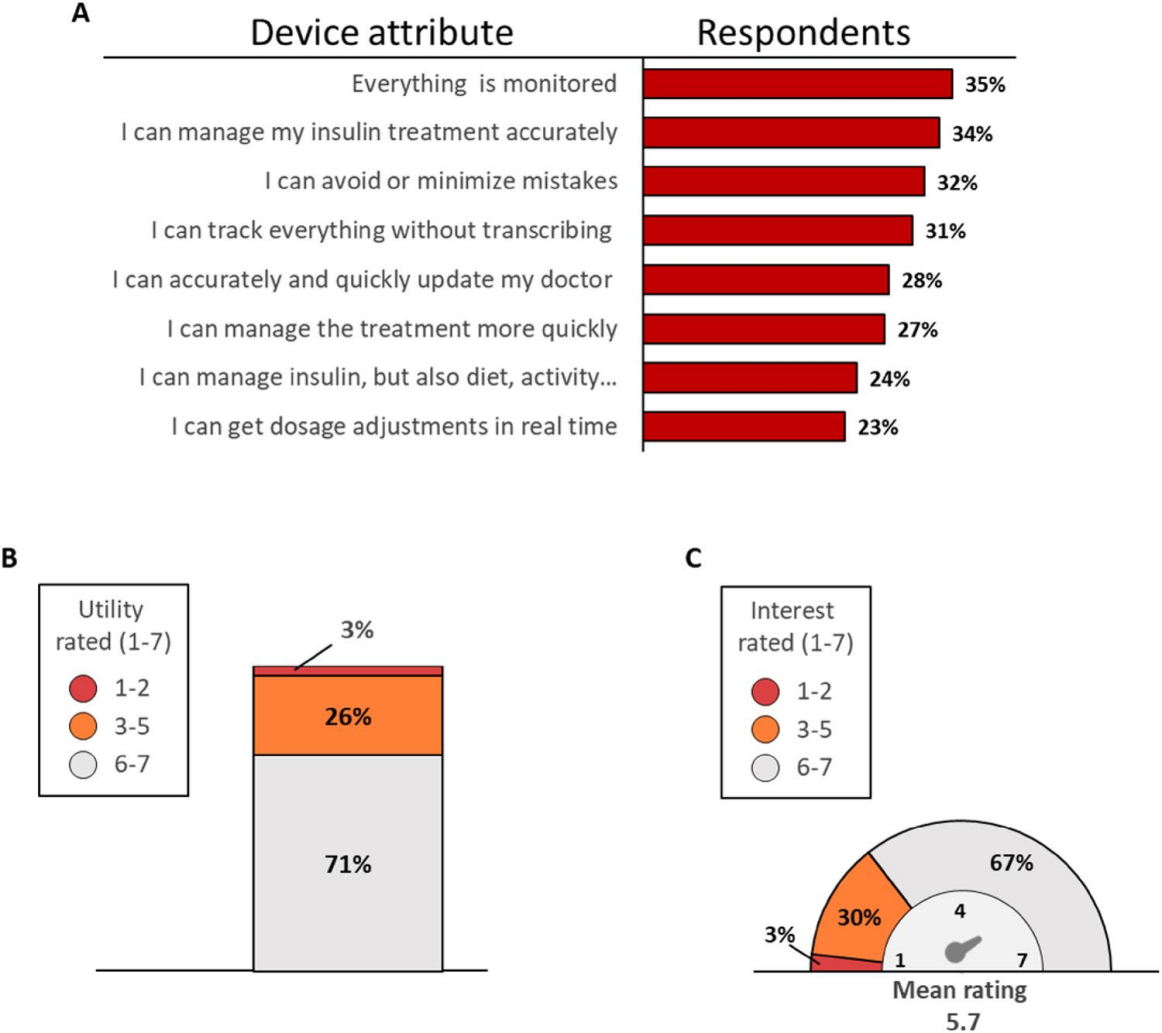
Comparison of the attributes of the patient’s current insulin pen with those of a new connected device. **A** The percentage of patients who selected these attributes when asked to choose up to 3 attributes; **B** Perceived utility of connected pen attributes (Question: “How useful do you think a connection-enabled insulin pen as described would be: rating from 1, not useful, to 7, extremely useful?”); **C** Interest in trying a connection-enabled insulin pen (Question: “How interested would you be in trying a connection-enabled insulin pen as described: rating from 1, not useful, to 7, extremely useful?”)

After the presentation of a video describing the attributes of a connected insulin injection device, patients were asked to rate the attributes. The highest ratings were for comprehensive monitoring capability, accurate insulin management, and avoiding mistakes (Fig. 1A); moreover, 71% of patients rated the new proposal “very useful” or “extremely useful” (Fig. 1B), and when asked about their interest in trying a connected device with the described attributes, two thirds of patients responded that they were “very interested” or “extremely interested” (Fig. 1C).

## Discussion

The American Diabetes Association and the European Association for the Study of Diabetes have identified patients’ choice as an important factor influencing medication decisions [26]. We have surveyed patients’ perceptions of insulin therapy in diabetes self-management to understand their unmet needs. Major patient concerns included the high cognitive burden associated with self-administration. We also assessed their impression of the potential of connection-enabled insulin devices to facilitate their diabetes care. The concept of a connected insulin pen was assessed favorably, compared to their non-connected pens. Findings suggest that connected insulin devices may improve patient experience with insulin therapy.

We assessed adherence to insulin treatment in this cohort of patients using non-connected insulin devices and found that more than a quarter of respondents had low adherence to therapy. This finding is consistent with the results of a patient preference survey of bolus insulin dose timing conducted in patients with T2DM that also used the MMAS-8 scale and reported poor adherence in approximately 24% of respondents [27].

Our findings are in line with those of a survey by Boye et al. [11], that assessed perceptions of injectable therapy among 504 patients with T2DM in the UK and US, showing that the most important characteristics of injectable medication were confidence in administering the correct dose (59.5%), ease of selecting the correct dose (53.2%), and overall ease of using the injection device (47.4%).

A discrete choice experiment conducted among 540 adult patients with T1DM or T2DM in the UK and US assessed patients’ preferences for a connected insulin device over non-connected devices and the relative importance that patients place on attributes of connected insulin devices [28]. This study also revealed that patients assign a high relative importance to device attributes providing support for calculating doses and automatic transfer of blood glucose data (i.e., connectivity), and that advanced systems with either a connected smart pen or SmartButton were of high interest. A discrete choice experiment on injectable treatments for T2DM conducted in Italian patients revealed a preference for simple treatment regimens [29].

Other recent developments that may contribute to simplifying diabetes treatment include ultra-long-acting basal insulin that allows once-weekly administration, and rapid-acting insulin that can simplify the timing of bolus dosing by eliminating the need for carefully timing of bolus doses before meals.

### Study limitations

Some of the problems raised by our survey involve subjective issues with the level of inconvenience or complexity of the treatment regimen, rather than clinical outcomes; however, ease of use and convenience can have a strong impact on outcomes by improving adherence and by allowing more patients to administer insulin correctly. Moreover, we did not have access to data on the levels of glycated hemoglobin or other information about long-term blood glucose control that would have been useful to compare with the MMAS-8 adherence scores. It should also be considered that people with diabetes were recruited based on self-reported diagnoses without clinical confirmation and were identified through a convenience sample drawn from an opt-in panel of individuals who signed up to participate in healthcare research studies; therefore, the generalizability of the findings remains limited.

## Conclusions

This study on more than 200 patients provides numerous inputs for future research. The cognitive burden associated with self-management of diabetes therapy drives preferences for advanced insulin delivery systems. When comparing the attributes of a non-connected insulin pen with those of a new connected device, most patients rated the new proposal “very useful”. Real-life studies about smart pens are needed to evaluate benefits in terms of clinical outcomes, quality of life, and treatment satisfaction.

## Supplementary Information

Below is the link to the electronic supplementary material.

Supplementary file1 (PDF 1546 kb)

Supplementary file2 (PDF 94 kb)

## Footnotes

1 The MMAS-8 Scale, content, name, and trademarks are protected by US copyright and trademark laws. Permission for use of the scale and its coding is required. A license agreement is available from MMAR, LLC., Donald E. Morisky, ScD, ScM, MSPH, 294 Lindura Ct., USA; donald.morisky@moriskyscale.com.
