# Supplementary material for: Patient perceptions of insulin therapy in diabetes self-management with insulin injection devices
Source: Acta Diabetol. 2023 Feb 25;60(5):705–10. doi: 10.1007/s00592-023-02054-7 (PMC10063495; doi:10.1007/s00592-023-02054-7)
Supplement: Supplementary file 1 — Supplementary file1 (PDF 1546 kb) [file 592_2023_2054_MOESM1_ESM.pdf]

Supplemental Tab 1 Survey designed to investigate patients' perceptions of daily diabetes management and insulin therapy

QUESTIONNAIRE					
Q1.	SINGLE ANSWER<br>How often do you usually measure your blood sugar?				
1	4 or more times a day				
2	2 or 3 times a day				
3	Once a day				
4	More than once a week, but less than once a day				
5	Once a week or less				
Q2.	SINGLE ANSWER<br>What method do you currently use to measure blood sugar? Please select an option.				
Q3.	NUMERIC ANSWER<br>How many insulin injections do you make in a day?				
	SHOW ONLY IF CODE 3 FROM S9<br>|__|__| dosing per day in conjunction with meals or snacks				RANGES 1-10
	SHOW ALL<br>|__|__| dosing per day away from meals				RANGES 1-2
D5.1	SINGLE ANSWER<br>She..				
1.	Do you follow a specific diet for your diabetes?	a- Yes, regularly	b- Yes, occasionally	c-No	
2.	Do you do activity?	a- Yes, regularly	b- Yes, occasionally	c-No	
D5.2	SINGLE ANSWER<br>How would you describe your lifestyle or typical day?				
1.	Regular, without many unexpected events				
2.	Frantic, I'm always in a rush and/or with many unexpected events				
D6.1	MULTIPLE ANSWER<br>RANDOM – CODE 4 and 5 POSITION FIXED<br>How do you currently record your insulin diary? That is, how it keeps track of the insulin administrations it carries out during the day, times, hours, etc				
1.	On a paper diary, given by the doctor or downloaded from the internet				
2.	Spreadsheet on the computer				
3.	Apps on your smartphone				
4.	Other (SPECIFY)				
5.	I DO NOT record/note insulin administrations				

D6.2	IF CODE 5 at D6.1<br>MULTIPLE ANSWER<br>RANDOM<br>Why don't you record your insulin diary, i.e. you don't keep track of the insulin doses that you administer during the day?						
	I remember it, there's no need to take note						
	I have no time						
	It's not useful/I find it useless						
	It's too challenging						
	Other (specify)						
Q7.	Some people have experienced problems with their medication behavior and we are interested in hearing about your experience. There are no right or wrong answers. Answer each question based on your personal experience with the insulin you take.						
1.	Do you sometimes forget to take your insulin?				No	Yup	
2.	Sometimes people don't take their medicines for reasons other than forgetfulness. Looking back over the past two weeks, have there been any days when you haven't taken your insulin?				No	Yup	
3.	Have you ever decreased your dose or stopped taking your insulin without telling your doctor because you felt worse when taking it?				No	Yup	
4.	When you travel or leave home, do you sometimes forget to take your insulin with you?				No	Yup	
5.	Did you take your insulin yesterday?				No	Yup	
6.	When you feel your symptoms are under control, do you sometimes stop taking your insulin?				No	Yup	
7.	Taking insulin every day is a real inconvenience for some people. Do you ever feel annoyed that you have to stick to your treatment plan?				No	Yup	
8.	How often do you have trouble remembering to take your insulin?	Never	Rarely	Sometimes	Often	All time	
D7.1	ASK IF "YES" at CODE 1 in D7<br>NUMERIC<br>You said that sometimes you forgets to take her insulin. On average how many times does this happen in a week?						
	I__I__I times a week						
D7.2	ASK IF "YES" at CODE 1 in D7<br>NUMERIC<br>You said that in the last two weeks there have been days when you haven't taken your insulin. How many times has this happened?						
	I__I__I times in the last two weeks						
D7bis	SCALE 1-7 – SINGLES PER ITEM<br>RANDOM<br>Thinking about your personal experience of diabetes care, we ask you to tell us how often you...						
1.	skipping appointments with my doctor/diabetologist	a-often	b SOMETIMES	c-NEVER			
2.	forget data about my diabetes, such as the value of the last blood sugar measurement (blood sugar value)	a-often	b SOMETIMES	c-NEVER			

3.	forget the number of units of my last insulin injection	a-often	b SOMETIMES	c-NEVER	
4.	forget the time of my last insulin injection				
5.	think that the management of my insulin therapy is complex	a-often	b SOMETIMES	c-NEVER	
D7.bis_ 1	ASK IF OFTEN" or "SOMETIMES" AT ITEM 3 in D7bis<br>NUMERIC<br>Think about a typical week, how many times do you **forget the number of units of the last administration of insulin** ?				
	I__I__I times a week				
D7.bis_ 2	ASK IF OFTEN" or "SOMETIMES" AT ITEM 4 in D7bis<br>NUMERIC<br>Think about a typical week, how many times do you **forget the time of the last insulin administration** ?				
	I__I__I times a week				
Q8.	ASK IF "YES" at CODE 1 or 2 or 3 or 4 or 6 or "NO" at CODE 5 in D7<br>MULTIPLE ANSWER<br>RANDOM<br>You said you happens to not take her insulin. In your opinion, why does it happen that you does not take insulin? Select all the answers you deem appropriate				
	Sometimes I forget about therapy...				
1.	When there is no one who reminds me				
2.	When there is no one who practically helps me to make/prepare it				
3.	Because it's inconvenient to take everything you need out of the house				
4.	Because I have difficulty complying with hiring rules (hours, specific moments of the day...)				
5.	Because there is a need for too much precision and I can't do it				
6.	Because it is easy to get confused if you have to manage so much information (dosage, time, date...)				
7.	Because I find the way I have to take it uncomfortable				
8.	Because I don't always understand the dosage/dosage adjustment				
9.	When I'm better				
10.	When it makes me feel worse				
11.	Other (SPECIFY) ANCHOR				
Q9.	ASK IF CODE 1 OR 2 (OFTEN or SOMETIMES) AT D7.2_2 AND/OR D7.2_3 AND/OR 7.2_4<br>MULTIPLE ANSWER<br>RANDOM<br>You said that you sometimes forget data about your diabetes, such as your last blood sugar level or your last insulin dosage or timing. In your opinion, why do you struggle to remember these aspects?				
1.	The data are too complex				
2.	There are too many things to record				
3.	I should always have my diary close at hand				
4.	I can't remember all the values				
5.	I'm not interested in remembering this data				
6.	Other (SPECIFY) ANCHOR				
d	SCALE 1-7<br>In general, how satisfied are you with your values and with how your diabetes is going? Please answer on a scale of 1 to 7, where 1=Not at all difficult and 7=Extremely difficult.				
	Not at all satisfied. Extremely satisfied 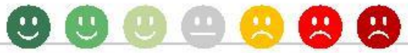				

d	SCALE 1-7<br>How worried are you about having LOW blood glucose levels? Please answer on a scale of 1 to 7, where 1=Not at all difficult and 7=Extremely difficult.	
	Not at all - Extremely	
d	SCALE 1-7<br>How worried are you about having HIGH blood glucose levels? Please answer on a scale of 1 to 7, where 1=Not at all difficult and 7=Extremely difficult.	
	Not at all - Extremely	
Q10.	SCALE 1-7<br>In general, how difficult is it for you to continue the insulin therapy you are taking? Please answer on a scale of 1 to 7, where 1=Not at all difficult and 7=Extremely difficult.	
	Not at all difficult - Extremely difficult	
Q11.	SCALE 1-7 – SINGLES PER ITEM<br>And specifically how satisfied are you?<br>Please answer using a scale from 1 to 7, where 1=Not at all satisfied and 7=extremely satisfied.	
	Not at all satisfied - Extremely satisfied	
1.	With the insulin therapy you are taking	
2.		
Q12.	ASK IF CODE 1 OR 2 OR 3 AT D11_1 OR D11_2<br>MULTIPLE ANSWER<br>RANDOM - MAX 3<br>What is not satisfying about the insulin therapy you are taking? Select the 3 factors that generate the greatest dissatisfaction today	
1.	I have to measure my blood sugar several times/too many times a day	
2.	I have to monitor and track blood sugar values	
3.	I have to vary the dose of insulin according to my values / It is difficult to understand the dosage I have to take each time	
4.	I have to remember to bring therapy with me if I'm out	
5.	I feel watched if I have to take insulin outside the home	
6.	It's a real discomfort if I'm away from home especially in my free time when I go out with people or go to clubs	
7.	It is difficult to keep track of time, date, dosage in a timely manner	
8.	The paper diary/diary is really a burden/encumbrance	
9.	I don't always remember how much insulin I take each day	
10.	It takes too much precision and I'm not a precise person	
11.	I am unable to report all the information on blood sugar and insulin to my doctor/diabetes specialist	
12.	Other (SPECIFY) ANCHOR	
Q14.	SCALE 1-7 – SINGLES PER ITEM<br>RANDOM<br>Thinking about the commitment that managing your diabetes means to you today, how important do you think it is to find a better way to...	

	Use a scale from 1 to 7 where 1=Not important at all and 7=Very important.	
	Not important at all Very important	
	It is important for me to find a better way to...	
1.	Administer insulin at the right time	
2.	Administer the correct amount of insulin	
3.	Communicate my data to my doctor/diabetologist	
4.	Correctly and regularly record the doses of insulin administered	
5.	Monitor my blood sugar values	
6.	Stay in the correct blood sugar ranges	
7.	Have a healthy diet/maintain a healthy weight	
Q15.	ASK IF D14 AT LEAST ONE ITEM > 2 STARS<br>MULTIPLE ANSWER<br>RANDOM<br>these improvements would allow it to...<br>Indicate a maximum of three answers.	
1.	Manage your therapy more accurately	
2.	Improve therapy times / manage measurement and intake phases faster	
3.	Keep track of everything without having to transcribe every time	
4.	Update my doctor accurately and quickly	
5.	Manage everything not just aspects related to insulin intake: blood sugar information, insulin doses, my diet, my physical activity program...	
6.	Have everything monitored/controlled	
7.	Have real-time dosage adjustments	
8.	Avoid or minimize errors/oversights	
9.	Other (SPECIFY) ANCHOR	
INTRO:	We will now show you the description of a possible new connection-enabled insulin pen:<br>INSERT DESCRIPTION	
Q16.	MULTIPLE ANSWER<br>SCALE 1-7 – SINGLES PER ITEM<br>RANDOM<br>How much do you agree or disagree with the following information regarding this possible change. Please use a scale from 1 to 5 where 1=Strongly disagree and 5=Strongly agree.	
	Strongly disagree Strongly agree	
	I think a connect-enabled insulin pen could...	
1.	Making my diabetes management easier	
2.	Allow me to have everything "recorded" in real time	
3.	Reduce the time I spend managing my diabetes	
4.	Allowing me to have all my diabetes data in “one place” would streamline my efforts	
5.	Being complex and that worries me	
6.	Cost me more time if difficulties arise (e.g. connection problems, ...)	
7.	Help me verify the actual insulin intake	
8.	Let me define the dosage adjustment	
9.	Have data privacy concerns	
10.	Be complex in its use	
11.	Being not ideal for me: it scares me to rely on digital technologies	
Q17.	MULTIPLE ANSWER	

	RANDOM<br>This new, connected-enabled insulin pen would mainly allow you to…<br>Please indicate up to three answers.	
1.	Manage your therapy more accurately	
2.	Improve therapy times / manage measurement and intake phases faster	
3.	Keep track of everything without having to transcribe every time	
4.	Update my doctor accurately and quickly	
5.	Manage everything not just aspects related to insulin intake: blood sugar information, insulin doses, my diet, my physical activity program…	
6.	Have everything monitored/controlled	
7.	Have real-time dosage adjustments	
8.	Avoid or minimize errors/oversights	
9.	Other (SPECIFY) ANCHOR	
Q18.	SCALE 1-7<br>In general, how useful would you find a connectable insulin pen as described? Use a scale from 1 to 7 where 1=Not at all helpful and 7=Extremely helpful.	
	Not at all useful. Extremely useful	
Q19.	SCALE 1-7 – SINGLES PER ITEM<br>And specifically how useful do you think the connection-enabled insulin pen as described can be…<br>Use a scale from 1 to 7 where 1=Not at all useful and 7=Extremely useful.	
	Not at all useful. Extremely useful	
1.	Suggest the insulin dose to be taken based on the blood glucose data automatically transmitted via the App	
2.	Automatically transfer dosage data so that you can view it on your smartphone	
D20.	SCALE 1-7<br>How interested would you be to try a connect-enabled insulin pen as described? Please answer on a scale of 1 to 7, where 1=Not at all interested and 7=Extremely interested.	
	Not at all interested Extremely interested	
Q21.	ASK IF CODE 3 OR 4 OR 5 AT D20<br>OPEN<br>What would a connection-enabled insulin pen as described make you feel? Would you try it if…	
Q22.	SINGLE ANSWER<br>Do you or have you ever used apps for smartphones or electronic devices to manage your therapy (e.g. to remember to take it correctly in therapy)?	
1.	I'm currently using it	
2.	I used to use it in the past	
3.	No	
Q23.	ASK IF CODE 1 OR 2 AT D22<br>SCALE 1-7 – SINGLES PER ITEM	

	RANDOM<br>Thinking about the use of technology in the management of your diabetes, how much do you agree with the following statements? Please use a scale from 1 to 5 where 1=Strongly disagree and 5=Strongly agree.	
	Strongly disagree 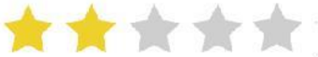 Strongly agree	
	The technologies ….	
1.	It has made my life better	
2.	It made my life easier	
3.	It has helped me get better/healthier	
4.	It has more pros than cons for managing my diabetes	
