# Supplementary material for: Patient perceptions of insulin therapy in diabetes self-management with insulin injection devices
Source: Acta Diabetol. 2023 Feb 25;60(5):705–10. doi: 10.1007/s00592-023-02054-7 (PMC10063495; doi:10.1007/s00592-023-02054-7)
Supplement: Supplementary file 2 — Supplementary file2 (PDF 94 kb) [file 592_2023_2054_MOESM2_ESM.pdf]

Supplemental Tab 2 Questions in the Morisky Medication Adherence Scale[a] [1-4]

You indicated that you are taking medication(s) for your diabetes. Individuals have identified several issues regarding their medication-taking behavior, and we are interested in your experiences. There is no right or wrong answer. Please answer each question based on your personal experience with your [health concern] medication.

	**Yes**	**No**
1. Do you sometimes forget to take your insulin?		
2. People sometimes miss taking their medications for reasons other than forgetting. Thinking over the past two weeks, were there any days when you did not take your insulin?		
3. Have you ever cut back or stopped taking your insulin without telling your doctor, because you felt worse when you took it?		
4. When you travel or leave home, do you sometimes forget to bring along your insulin?		
5. Did you take your insulin yesterday?		
6. When you feel like your diabetes is under control, do you sometimes stop taking your insulin?		
7. Taking medications every day is a real inconvenience for some people. Do you ever feel hassled about sticking to your diabetes treatment plan?		

8. How often do you have difficulty remembering to take all your medication?

(Please circle your answer below)

Never/Rarely	a
Once in a while	b
Sometimes	c
Usually	d
All the time	e

[a] The MMAS-8 Scale, content, name, and trademarks are protected by US copyright and trademark laws. Permission for use of the scale and its coding is required. A license agreement is available from MMAR, LLC., Donald E. Morisky, ScD, ScM, MSPH, 294 Lindura Ct., USA; donald.morisky@moriskyscale.com.

1. Morisky DE, Ang A, Krousel-Wood M, Ward HJ (2008) Predictive validity of a medication adherence measure in an outpatient setting. J Clin Hypertens 10:348–354. https://doi.org/10.1111/j.1751-7176.2008.07572.x
2. Berlowitz DR, Foy CG, Kazis LE, et al (2017) Effect of Intensive Blood-Pressure Treatment on Patient-Reported Outcomes. N Engl J Med 377:733–744. https://doi.org/10.1056/NEJMoa1611179
3. Bress AP, Bellows BK, King JB, et al (2017) Cost-Effectiveness of Intensive versus Standard Blood-Pressure Control. N Engl J Med 377:745–755. https://doi.org/10.1056/NEJMsa1616035
4. Fabbrini G, Abbruzzese G, Barone P, et al (2013) Adherence to anti-Parkinson drug therapy in the "REASON" sample of Italian patients with Parkinson's disease: the linguistic validation of the Italian version of the "Morisky Medical Adherence Scale-8 items." Neurol Sci 34:2015–2022. https://doi.org/10.1007/s10072-013-1438-1
